# Exploring children’s stigmatisation of AIDS-affected children in Zimbabwe through drawings and stories

**DOI:** 10.1016/j.socscimed.2010.05.028

**Published:** 2010-09

**Authors:** Catherine Campbell, Morten Skovdal, Zivai Mupambireyi, Simon Gregson

**Affiliations:** a Institute of Social Psychology, London School of Economics and Political Science, Houghton Street, London WC2A 2AE, UK; b Biomedical Research and Training Institute, Harare, Zimbabwe; c Department of Infectious Disease Epidemiology, Imperial College London, London, UK

**Keywords:** Zimbabwe, AIDS, Stigma, Children, Psychosocial well-being, Drawings, Social representations, Africa

## Abstract

AIDS-related stigma is a major contributor to the health and psychosocial well-being of children affected by AIDS. Whilst it is often suggested that AIDS-affected children may be stigmatised by other children, to date no research focuses specifically on child-on-child stigma. Using social representations theory, we explore how Zimbabwean children represent AIDS-affected peers, examining (i) whether or not they stigmatise, (ii) the forms stigma takes, and (iii) the existence of non-stigmatising representations that might serve as resources for stigma-reduction interventions. Our interest in identifying both stigmatising and non-stigmatising representations is informed by a theory of change which accords a central role to community-level debate and dialogue in challenging and reframing stigmatising representations. In late 2008, 50 children (aged 10–12) were asked to “draw a picture of a child whose family has been affected by AIDS in any way”, and to write short stories about their drawings. Thematic analysis of stories and drawings revealed frequent references to stigmatisation of AIDS-affected children – with other children refusing to play with them, generally keeping their distance and bullying them. However children also frequently showed a degree of empathy and respect for AIDS-affected children’s caring roles and for their love and concern for their AIDS-infected parents. We argue that a key strategy for stigma-reduction interventions is to open up social spaces in which group members (in this case children) can identify the diverse and contradictory ways they view a stigmatised out-group, providing opportunities for them to exercise agency in collectively challenging and renegotiating negative representations. Contrary to the common view that drawings enable children to achieve greater emotional expression than written stories, our children’s drawings tended to be comparatively stereotypical and normative. It was in written stories that children most eloquently expressed meanings and emotions, and an awareness of the complexity of the scenarios they portrayed.

## Introduction

Children are increasingly targeted in HIV/AIDS programming due to the growing number of HIV positive children and growing recognition of children’s role in responding to the epidemic, particularly as supportive and caring actors (Skovdal, 2010; Skovdal, Ogutu, Aoro, & Campbell, 2009). Children’s stigmatisation of each other is a vitally important and under-researched area (Deacon & Stephney, 2007). We use children’s drawings and stories to investigate their representations of AIDS-affected children. Viewing children as competent social actors, constantly engaged in the construction and reconstruction of the social representations that give meaning to their experience, we aim to highlight the diversity and complexity of children’s representations. We do this in the interest of informing stigma-reduction programmes that seek to acknowledge and strengthen the existence of positive and empowering representations of socially excluded groups that may often exist alongside stigmatising ones.

Goffman (1963: 13) defines stigma as “an attribute that is significantly discrediting, which in the eyes of society, serves to reduce the person who possesses it”. Building on this, we draw on Dovidio, Major, and Crocker’s (2003) conceptualisation of stigma as the blend of affective, cognitive and behavioural responses to those bearing ‘discrediting attributes’, constructed within a collectively negotiated body of social knowledge or representational field. In this paper, we seek to characterise the representational field in which children’s responses to their AIDS-affected peers are negotiated.

The form and content of stigma varies across cultures and socio-economic contexts (Deacon & Stephney, 2007; Norman, Abreu, Candelaria, & Sala, 2009). However, whatever shape it takes, stigma presents a major barrier to effective HIV/AIDS management. Ban Ki-moon (2008: 1), UN secretary general, attributes the continuing and devastating impact of AIDS to stigma, “the silent killer of AIDS”, identifying it as the most important barrier to public action. Numerous studies testify to its devastating effects. In South Africa for example, Campbell, Nair, Maimane, and Sibiya (2008) found that fear of disclosing HIV status hindered people’s access to the already limited care or support that was available. Stigma has been identified as the chief barrier to uptake of antiretroviral medication and adherence for people living with HIV and AIDS (PLWHA) (Weiser et al., 2003).

Although the HIV prevalence in Zimbabwe has fallen in recent years from 25.3% in 1997 to 13.7% in 2009 (Zimbabwe Ministry of Health and Child Welfare, 2009), numerous challenges continue to face the children whose parents develop AIDS and/or become orphaned – one of which is stigma. Using drawings and stories, we explore how Zimbabwean children represent AIDS-affected peers, in the interests of examining (i) whether or not they stigmatise, (ii) what forms stigma might take, (iii) the existence of non-stigmatising representations that might serve as a resource for stigma-reduction interventions.

## Children’s attitudes to PLWHAs

Children may express, understand and respond to AIDS-related stigma differently from adults (Deacon & Stephney, 2007). Little is known about how children stigmatise AIDS-affected *children*. However, a growing body of literature has explored children’s attitudes to PLWHA *in general* – and we take this literature as our starting point. Studies have highlighted the relationship between HIV/AIDS knowledge and children’s attitudes towards PLWHA. In Mali for example, Castle (2004) showed that school children’s stigmatisation of PLWHA was associated with misconceptions that AIDS could be transmitted through casual social contact – views also held by community elders, parents and school teachers. Letamo (2004) found children in Botswana to fear social contact with PLWHA, with children saying that HIV positive teachers should not be allowed to teach. However, these children were still willing to care for sick relatives and were more likely to stigmatise PLWHA who were not close relatives. In South Africa, Maughan-Brown (2006) found the prevalence of negative judgements and fear of HIV infection were greater than negative behavioural intentions towards PLWHA. In Swaziland, Buseh, Park, Stevens, McElmurry, and Kelber (2006) found gender to be a significant factor in predicting stigma. Boys were less sympathetic to PLWHA than girls, showing higher levels of stigmatising attitudes. Peer influence, conceptualised as the number of discussions children had with friends about HIV/AIDS, was associated with lower stigma levels (ibid.) – suggesting that interventions should create social spaces facilitating dialogue about AIDS and renegotiation of gender roles.

Children not only stigmatise, but can also be stigmatised against, often undermining their psychosocial well-being (Cluver & Gardner, 2007; Makame, Ani, & Grantham-McGregor, 2002). Studies in South Africa explore the sources and outcomes of stigma, and its impacts on the mental health of AIDS-affected children. Strode and Barrett (2001) document the stigmatisation of AIDS-affected children by their extended families, communities, school peers and health and social services, outlining some of its consequences, including reduced access to care and support, increased vulnerability to poverty and exploitation, fear of disclosing HIV status, feelings of powerlessness and emotional pain. Bullying and the stigmatisation of AIDS-affected children have been associated with psychosocial distress (Cluver & Orkin, 2009). Teachers have observed bullying of AIDS-affected children by other children (Baggaley, Sulwe, Chilala, & Mashambe, 1999) and Hejoaka’s (2009) study of mothers with AIDS-affected children in Burkino Faso highlighted children’s fear that if their peers knew about their HIV status they would not play with them.

Whilst references to child-on-child stigma have been made in passing, no studies have explicitly investigated it. To address this gap we examine children’s representations of AIDS-affected children to develop our understandings of what drives and mitigates AIDS-related stigma as well as informing the on-going challenge of developing effective stigma-reduction programmes among children. We conclude by discussing our findings in relation to children’s capacities to exercise agency through resisting the internalisation of stigmatising social representations, and showing empathy and solidarity for AIDS-affected children.

## Conceptual framework

Social representations theory (SRT) (cf. Moscovici, 1984) provides a productive lens for examining the sense people make of their social worlds. Moscovici (1973: xiii) defines social representations as “systems of values, ideas and practices with a two-fold function; first, to establish an order which will enable individuals to orientate themselves in their material and social world and to master it; and secondly to enable communication to take place among the members of a community by providing them with a code for social exchange and for naming and classifying the various aspects of their world and their individual and group history.” Common-sense knowledge is a product of local context. Social representations are dynamic, rather than static, systems of social knowledge. They are constantly negotiated and renegotiated in the course of daily interactions between people, groups and institutions in particular historical settings through dialogue and communication as people go about their daily lives. The process of representation involves a constant tension between stability (the possibility of reproducing traditional understandings) and change (the possibility of transforming these through constructing novel ways of making sense of the world). SRT’s emphasis on the potential for representations to change is particularly relevant to our interest in the possibility of challenging and transforming stigmatising representations through stigma-reduction programmes.

SRT has been used to explore stigma in relation to the ‘othering’ of the mentally ill, notably in Jodelet’s (1991) classic study of interactions between the mentally ill and their carers (French villagers paid to lodge psychiatric patients in their homes). Representations of the mentally ill as ‘other’ shaped the distancing and discriminatory rituals and practices that shaped carers’ interactions with patients. According to Jodelet, carers constructed these distancing representations and practices as symbolic boundaries between themselves and what they experienced as the unpredictable, strange or potentially frightening behaviour of the patients. Representational boundaries give individuals a sense of comfort and safety in a deeply unpredictable social world, in which human beings must constantly construct psychological defences against the threat of chaos and disruption. Stigmatising representations also helped the carers cope with the ‘identity threat’ posed by the nagging and terrible possibility that they too might one day be vulnerable to a strange and disturbing affliction (Joffe, 1999). Crawford (1994) argues that AIDS, associated with the taboo issues of death and sexuality, constitutes a similar ‘symbolic threat’. AIDS stigma is driven as much by peoples’ dread of ‘symbolic contagion’ as of physical contagion, with the existence of AIDS exacerbating the constant unconscious fear of death and disruption that characterise human existence (Treichler, 1999).

Various dimensions of AIDS stigma are shaped by wider power inequalities (Parker & Aggleton, 2003). These dimensions may include the fear of physical contagion (instrumental stigma), the association of AIDS with immorality and shame (symbolic stigma), the idea that AIDS-affected people do not deserve access to health care or services (resource-based stigma) and the stigmatisation of people through their association with diseased relatives or friends (courtesy stigma) (Maughan-Brown, 2006).

## Methodology

Ethical approval was granted by the Medical Research Council of Zimbabwe and a research committee at the London School of Economics. We followed UNICEF (2002) guidelines on undertaking research with children and written consent (for their participation and the publication of their stories and drawings) was obtained from all participating children and their parents/guardians.

### Study participants

Fifty Shona-speaking children aged 10–12 participated. Children of this age have not yet reached full sexual maturity, and most will not yet be sexually active. As a result, the sexual anxieties that drive AIDS stigma might take a rudimentary form relative to adults, making this age group particularly ripe for anti-stigma interventions *before* the emotions associated with sexual relations have fully consolidated. Furthermore, many children of this age have the skills and training to express ideas and feelings in writing as well as through drawings. Data were collected in September 2008, by the third author (ZM), a Shona-speaking Zimbabwean who is both an academic social psychologist and experienced social worker. Participants constituted a convenience sample, drawn from rural and urban areas in which she has lived and worked. Although participants were purposively selected to ensure an equal gender and rural/urban divide, no differences emerged in our analysis of the stories and writing produced by these groups, so we pooled the data for our analysis. Children were not selected on the basis of, or asked to declare, whether they themselves were living with HIV/AIDS or whether their own families were affected by HIV/AIDS.

### Data collection

We used an open-ended approach to elicit children’s own voices and perspectives (James & Prout, 1997). Besides being a fun participatory activity for children, drawings have traditionally been used by psychologists to explore the meanings children give to their emotional, mental and intellectual experiences (Backett-Milburn & McKie, 1999). Drawings encourage children to talk about their inner and social worlds, communicating thoughts and feelings they may find difficult to articulate in speech or writing due to their limited vocabulary and inability to articulate their emotions verbally (Pridmore & Bendelow, 1995). However, Pridmore and Bendelow (1995: 219) argue that an ‘imaginative combination of drawing and writing’ should be used to counterbalance the strengths and limitations of drawings alone. This combination of methods also allowed less literate children to focus their energies on drawing if they felt less comfortable writing.

Children were asked to produce the drawings and stories individually in their own homes. After explaining the study and going through formal consent procedures, the researcher asked guardians and others to vacate the room whilst drawing and writing were in progress. Children were asked to “draw a picture of a child whose family has been affected by AIDS in any way”. No further prompts were given, so children were free to express their views – either from their own experience, or from third person or fictitious stories. Thereafter children were asked to write short stories about the drawings. In eight instances children with limited literacy opted to narrate their stories verbally to the researcher who transcribed them. This generated 50 drawings and 50 stories. There was great variation in the quality and length of drawings and stories, with the latter ranging from 50 to 500 words. Thirty-nine children chose to write in English, and eleven in Shona (later translated into English by the researcher).

### Data analysis

Given that social representations are properties of social groups rather than of individuals, we did not aim to make links between individual children’s accounts and their individualised personal experiences, but rather to map out the stock of symbolic resources used by our informants to make sense of the impact of the epidemic on children. The entire data corpus constitutes our unit of analysis, rather than individual children’s accounts. Furthermore, in line with our social representations approach, the aim of our data collection was to map out the diversity of responses in the data set, rather than to generate accounts of stereotypical attitudes held by all the research participants.

**Fig. 1:**
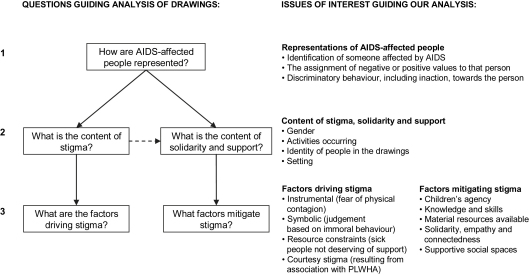
Three-step analysis of drawings.

Drawings and accompanying stories were analysed as a single unit. To minimise imposing the analysts’ own interpretations on the drawings, the story was read first to throw light on the content of the child’s written account of the picture. Against this background, each drawing was examined using a three-step analytical framework (see Fig. 1) which started by exploring how AIDS-affected children were represented in the drawing. An initial reading of the stories and preliminary viewing of drawings suggested that children’s representations could be classified as negative (indicating stigmatisation of the portrayed child), positive (indicating solidarity and support) or both. The second step therefore involved exploring the content (stigma and/or solidarity/support) of the drawing. The final step involved classifying any account that threw light on the social psychological factors driving or mitigating stigma, drawing on our conceptualisations of the ‘AIDS competent community’ (Campbell, Nair, Maimane, & Gibbs, 2009; Skovdal & Campbell, in press) to identify social psychological factors that might serve as resources for stigma-mitigation.

The five questions guiding the analysis of drawings are presented in Fig. 1 alongside examples of ‘issues of interest’ we looked for as we analysed them. Our identification of issues of interest was informed by an iterative process taking account of insights emerging from our literature review as well as our preliminary readings of the stories and drawings. We paid attention to the setting (e.g. community, hospital, homestead) as well as the activities carried out by people in the drawings.

Using Attride-Stirling’s (2001) thematic network analysis all written stories were read and re-read and topics emerging from drawings and stories were coded, leading to 32 codes. The frequency of each code across the entire data set is noted in Table 1, as is a list of the issues that were classified within each code. Following this process, codes were grouped together into basic themes, which were eventually clustered into three organising themes that highlight the representational field in which children's views and attitudes towards AIDS-affected children are negotiated. Our presentation of findings will reflect the structure of this coding framework (see Table 1).

## Findings

### Representations of AIDS

#### Children’s knowledge of AIDS

Children often had accurate factual knowledge. They knew AIDS could be transmitted through sex. Whilst some spoke of HIV being transmitted primarily amongst high risk groups (e.g. sex workers), others knew AIDS could affect anyone.

Nowadays there is a deadly disease called AIDS. This disease is only treated but not cured. Anyone can be infected – male, female, young, old, rich, poor, educated, uneducated from any country or any religion (Janet, urban).

They attributed their knowledge of HIV/AIDS to the attention AIDS receives in Zimbabwe.

AIDS is a sexually transmitted disease. Health workers, the radio and the television are always talking about AIDS (John, rural).

However, alongside accurate knowledge about transmission, many children also exhibited erroneous beliefs, often structured around an exaggerated fear of contagion. One commonly held belief was that eating with a PLWHA could lead to HIV transmission.

AIDS is prevented by not sharing razor blades, needles, syringes and by not having sexual intercourse. You must not eat in the same place with a person who has AIDS (James, rural).

These parents gave birth to twins. One of them was infected with AIDS. The other child got infected when the other one was coughing and when they were eating together (Tendary, boy, rural).

**Fig. 2:**
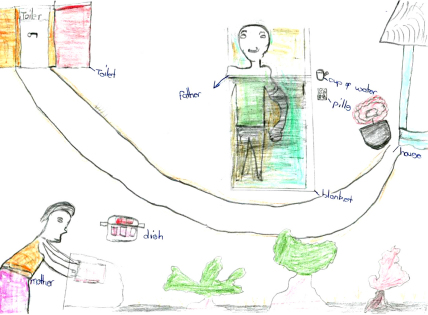
Person living with AIDS and on ARVs.

At the time of this study a minimal roll-out of antiretroviral drugs (ARVs) had just started. Five children spoke about ARVs and the types of care and support required for drugs to be effective, including care, love, and a balanced vitamin-rich diet. Two children drew ARVs next to a bedridden AIDS-affected guardian/parent and another family member engaged in caring or cooking food (Fig. 2).

We can help this girl by giving her tablets called ARVs. People with AIDS must eat a lot of vitamins. We should give this girl care and love (Martin, urban).

He is taking ARV pills. With ARVs he is getting stronger. These pills require you to eat a balanced diet (Matthew, rural).

#### Men as conduits of AIDS

Many children spoke of AIDS as a disease spread by ‘bad’ behaviour. Drinking and beer halls were seen as particular contributors here.

In the morning my father would go to the beer hall and drink beer and see his girlfriend. My father has too many girlfriends at the beer hall (Everline, rural).

This man was a drunkard. He was well-known in the whole city. Sometimes he slept with women who were married. He went to a VCT centre and was tested positive. He did not tell his wife. He spread the disease to the whole family (Tatenda, boy, rural).

Many children referred to men as more likely to engage in ‘bad’ behaviour, causing HIV to spread. Often such men were described as ‘old’, ‘fat’ or ‘rich’. Several spoke of younger girls sleeping with older men for gifts.

Louise lived with her mother. One day she was walking and saw a car. In the car was an old man. He said get into the car and she opened the door and got in. The man asked her to sleep with him at his house and she agreed. She was given sweets and chocolates. She was tempted with these since she was a little girl. Soon she fell sick and her mother took her to a VCT centre and she was found HIV positive (Chris, urban).

#### Impacts of AIDS

**Fig. 3:**
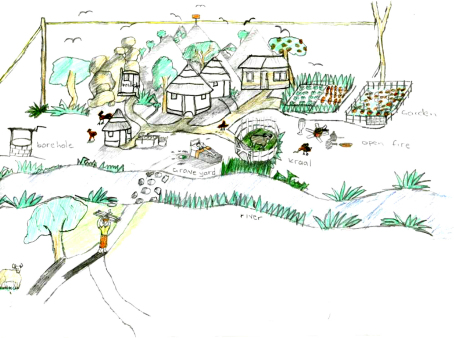
People bedridden from AIDS are close to death.

Children often mentioned death as an unavoidable outcome of HIV/AIDS. Some depicted grave-stones within homesteads. Whilst burying a family member within a homestead is not uncommon, one child illustrated the link between AIDS and death by depicting a bedridden person with AIDS near the graveyard of the homestead where the child lived (see Fig. 3).

**Fig. 4:**
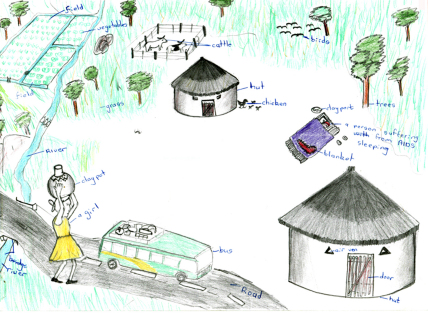
AIDS-affected child busy caring for sick parent.

**Fig. 5:**
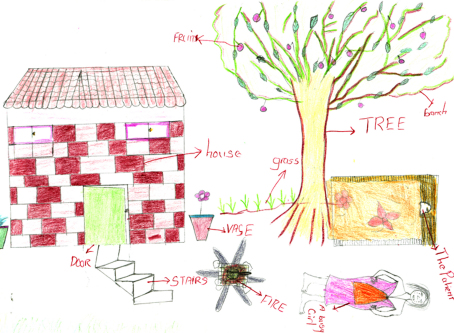
Drawing of an AIDS-affected child who is crying.

Aside from experiencing death of parents and loved ones, AIDS-affected children were represented as having seriously ill and bedridden parents for long periods. Nearly half the drawings depicted a bedridden person with AIDS, as Figs. 2–5 show. In addition to being described as bedridden and ‘useless’, people living with AIDS were often portrayed as weak, messy, malnourished and ill-looking.

He is sleeping outside. No one likes him. He is weak and tired. The bucket shows that he is just vomiting all the time (John, rural).

There was a girl, eleven years of age, that was born infected with HIV/AIDS. She has a small body. She was always sick. Her parents died when she was six years old […] She is a mess everywhere and always vomits. She was short, slim and black in complexion (Loyce, rural).

The terminal nature of AIDS, if left untreated, dominated many stories and some drawings, in addition to the economic and domestic unproductiveness of bedridden parents. Furthermore, children often presented *themselves* as knowledgeable and sympathetic to the circumstances of AIDS-affected children, but represented *other* children as having irrational fears of contagion.

### Representations of negative impacts of AIDS on children

Three themes dominated accounts of the negative impacts of AIDS on children.

#### A compromised childhood

AIDS-affected children were often represented as busy providing care and sustaining their households.

Alice spends most of the day cooking, washing, sweeping […] Alice’s mother will call Alice to come and help if she needs to go to the toilet. […] In the morning Alice will cook for her mother and she will bring her food and a bottle of water, a cup and some pills. She is always busy as a bee, she has no time to rest (Chipo, boy, urban).

Many informants said such children had few opportunities to enjoy being a child who attended school regularly, and played with friends. Some described AIDS-affected children as hard-working, with little time to rest. As Fig. 4 illustrates, some children drew the AIDS-affected child working in their homestead during the day, a time when children are usually in school. In a context where education is seen as the only route out of poverty, this is particularly significant.

She does not go to school or go and play with the other children because her work is more important than playing (Mark, rural).

Sometimes they [AIDS-affected children] do not have time to go to school because they need to care for a parent. At school they sit at the back of the classroom. The other children don’t get near them and this affects their learning. Also, they are thinking of their sick parent (Rufaro, girl, urban).

As the patched blanket in Fig. 4 symbolises, AIDS-affected children were depicted as living in poverty. Some informants drew AIDS-affected children with long hair (with stories explaining they could not afford hair cuts), patched clothing (unable to afford new clothes) and thin (unable to afford a good diet). Whilst most drawings and stories referred to a child living with an ailing parent and thus busy with caring duties, a few stories concerned an AIDS-affected child whose parents had died and who lived with relatives or other community members. Children without parents were occasionally described as abused and exploited due to the combined stigma of poverty, orphanhood and AIDS. Abuse included discriminating against orphaned children in the distribution of food and household chores, and failing to offer them the same support as non-orphaned children.

The parents of this child died of AIDS. This child is abused by everyone. Every person who passed through their place will shun the child. The relatives who used to stay with this child gave the child less food comparing with others. At times, this child would sleep outside. No one would buy clothes or shoes for this child. The child was told to do all the household chores. Some people would spit and laugh at this child because s/he is an orphan without shoes (Pamela, rural).

A few spoke of orphaned children as vulnerable to sexual abuse.

They lived happily (orphaned girl and uncle) until one day when the uncle came back from work. He asked her to pack his clothes. Whilst packing he grabbed her and raped her. This uncle is HIV positive. The uncle threatened her not to tell anyone (Mwaita, boy, urban).

#### Psychosocial distress

The perceived vulnerability of AIDS-affected children meant that many represented them as distressed and despairing (e.g. one child drew an AIDS-affected child holding her head) or crying (e.g. Fig. 5). AIDS was occasionally described in stories as a source of unhappiness, shattering futures.

Once upon a time, Robson was a happy child. But once this dreadful and incurable disease, HIV/AIDS, came into his life, his happiness was shattered. He had nothing to welcome in his life except a dark future. His goals and dreams were also blocked. The boy’s life was ruined (Fatima, boy, rural).

Aside from removing hopes for a bright future, AIDS-affected children were sometimes depicted as in a constant state of worry, fearing the death of parents or relatives living with AIDS.

The child is grieved because the grandmother and grandfather are suffering from AIDS, thinking that they might die (Mary, rural).

Children’s lack of friendship bonds was also seen as having a negative impact on their psychosocial well-being, leading to loneliness and isolation.

The girl is lonely, she doesn’t have time to share her thoughts and ideas with other girls (Carolyn, rural).

#### Ostracizing of AIDS-affected children

It was not only lack of time that prevented AIDS-affected children from developing friendships. Many informants said children were often afraid to play with AIDS-affected children. Some spoke of how children bullied and ostracised AIDS-affected children by teasing and calling them names.

The two children have no friends. It is because the other children run away from them. They think they will also get HIV from playing with them. These children, when they are free, play by themselves (Rufaro, girl, urban).

When she went to school her friends scolded and laughed at her. […] and others called her by names (Tatenda, boy, rural).

It was not only children that kept a distance from AIDS-affected children for fear of contracting HIV. Relatives and community members were also reported to mistreat AIDS-affected children.

Her relatives and friends started laughing at her. They thought if someone has got AIDS it will be the end of their life or if someone is taking care of someone with this deadly disease that person will also get infected, but that is not true […] her friends started teasing her and did not want to play or talk to her. She was now the saddest girl in the class. She asked for help from her relatives but they all turned her down (Josephine, rural).

The people of the community are not giving any support to this family. They don’t even go and see them because they also think that they will get the virus (Fatima, boy, rural).

These representations suggest that AIDS can have a profoundly negative impact on AIDS-affected children, who were portrayed as vulnerable to stigmatisation by other children, including fear of physical contact (instrumental stigma), having reduced access to community and peer support (resource-based stigma) and being tainted by their parent’s or relative’s ailment (courtesy stigma).

### Positive representations of AIDS-affected children

Social representations are seldom stereotypical or homogenous, reflecting the complexity of the meanings children give to complex situations. Several informants constructed complex and ambiguous representations reflecting both their dread of the plight of AIDS-affected children, but also a begrudging respect, even awe, and compassion for the enormity of their life challenges. In addition to representing AIDS-affected children as lonely, abused, stigmatised and sad, several informants also, or exclusively, depicted AIDS-affected children in a positive way.

#### Social support available to AIDS-affected children

**Fig. 6:**
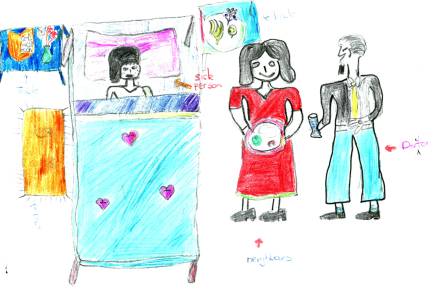
Drawing of neighbour and ‘doctor’ (community health worker) visiting an AIDS-infected parent.

Several referred to the support available to AIDS-affected children. They and their sick parents were said to benefit from support from neighbours, community health workers (see Fig. 6) and relatives who helped with nursing and caregiving.

When her father is sick relatives always come and visit. This makes him heal in short time. I have learnt that people who are HIV positive need love (Nyaradzo, girl, urban).

Mary gets support from community health workers. They always visit her and give her gloves, soap and food. I hope she will be able to keep on caring for her father (Florence, urban).

Some spoke of friendships that AIDS-affected children developed with other children and how they could take a break from caring activities to play with them. Friends were also mentioned as understanding and helpful.

After washing the plates he goes to play with his friends. Then he will come back and prepare lunch and talk to his sick father (Ngaavongwe, girl, urban).

Around ten o’clock some male neighbours come and wash her father […] she is never lonely because relatives and neighbours assist her […] She finds it difficult to care for her father, but other children often come to help (Edison, boy, urban).

She goes to her friend Caroline’s house and together they collect water […] Caroline understands the situation at Cellestine’s house so when they come back, she helps Cellestine doing all the house work. Surely a friend in need is a friend indeed. Caroline helps Pamela all the time […] Many things would be difficult for Cellestine, but with Caroline there things are much better (Janet, urban).

#### Caring children

Whilst our informants referred to the demanding nature of children’s caregiving activities, they did not depict AIDS-affected children as unwilling and ‘forced’ to care. Some spoke of children being motivated by love and concern for those living with AIDS, including concerns about their personal hygiene, diet and the availability of drugs.

Ellen has a good heart, she gives her father love and cooks for him everyday (Florance, urban).

She spends all day with her father. She tells him about school, encourages him to eat good foods and takes him for exercise so his bones will be strong (Matthew, rural).

Every day she wakes up and sweeps the yard and the house. After that she washes the plates and cooks porridge for her parents. When she has finished she washes her mother and her twin brother washes their father […] she always buys fruit for her parents, such as apples, bananas and oranges. She encourages them to eat them because they are good for human health (Lydia, urban).

**Fig. 7:**
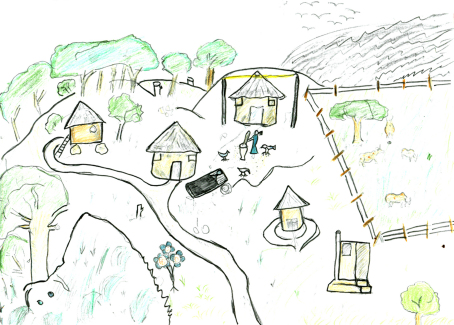
Child sustaining the household through care and domestic work.

Many informants referred to AIDS-affected children engaging in income-generating activities. As depicted in Figs. 2, 4 and 7, many children were depicted as having access to livelihood sustaining resources, including livestock, poultry and farmland. These helped children support bedridden and economically inactive parents.

Sometimes she went to the garden and started growing vegetables to sell so that they would have money to buy food and medicines for her parents (Josephine, rural).

#### Empathy and respect

Some informants spoke of AIDS-affected children with respect and empathy. Some even reported being envious of the agency of their caring peers, saying that caregiving children would be protected by God’s blessings. Even if they were not envious, they often used positive adjectives to describe AIDS-affected children.

I would like to be as helpful as Tafadawa and Tabudzwa are, as God will not let them grow without blessing (Lydia, urban).

Mary is a tolerant and compassionate girl who is loving to her father. She is an active girl. She is faithful as a dog. She believes that one day her father will be fine (William, rural).

The care and concern felt by many children was reflected in the advice some sought to give their AIDS-affected peers.

I would like to advise children that praying with a person with AIDS, sharing a bedroom, sharing toilets, helping that person to eat, playing games, washing his or her clothes, hugging that person, sitting next to that person or even sharing a plate with that person will not spread AIDS (Janet, urban).

Some children translated their admiration and respect into a narrative where the AIDS-affected child excelled in life and engaged in activities advocating for social change.

Kaski (AIDS-affected child) started her own anti-stigma programme. The Red Cross offered to pay her school fees. She was astonished by the number of people who liked the anti-stigma programme. Kaski was happy and people started to recognise her in the society. Two years later, Kaski passed her form six and went to university. She became a physician and was the first person to prevent stigma in the country (Tatenda, boy, rural).

## Discussion

We have examined how children represent AIDS and AIDS-affected children, to advance understandings of whether and how children stigmatise AIDS-affected peers. Our analysis of drawings and stories found the impact of AIDS on children was overwhelmingly presented as negative. Children were depicted as deprived, discriminated against, rejected, ‘children with no childhood’ and as children with reduced educational opportunities impacting negatively on their future life chances.

AIDS was represented as a deadly and contagious disease which could be transmitted through casual social contact such as handshakes or mixing with infected or affected people. These beliefs were said to result in the stigmatisation of AIDS-affected children by other children, often through bullying and a refusal to play with them. Fear of contagion has been observed amongst children in Burkina Faso (Hejoaka, 2009) and Puerto Rico (Gonzales-Rivera & Bauermeister, 2007), often attributed to poor understandings of HIV transmission (Maughan-Brown, 2006; Sallar, 2009). However many of our child informants had a sound and often sophisticated understanding of HIV transmission and management, suggesting that stigma was not always driven by factual ignorance. Furthermore many informants constructed more complex and ambiguous representations that included feelings of admiration, respect and empathy.

Although many children spoke of AIDS as something caused by socially condemned behaviours, the children of parents with AIDS were not themselves condemned as immoral by association. On the contrary, reflecting empathy for AIDS-affected children, our informants often emphasised the admirable efforts of AIDS-affected children as they provided care and support for ailing parents. These duties earned them respect from other children, reflected in the positive and admirable qualities they attributed to caregiving children. Whilst children have previously been observed to draw on representations of care and concern to resist the stigmatisation of adults living with HIV/AIDS (Bhana, 2008; Letamo, 2004), we can report on similar representations for AIDS-affected children. Furthermore, many children spoke of the support available to those affected by AIDS, including neighbours, relatives and the friends of AIDS-affected children.

Contrary to Buseh et al. (2006) who found that Swazi boys stigmatised PLWHA more than girls, we found no differences in the representations constructed by male and female informants. However, our findings did cast interesting light on children’s understandings of the role of gender in HIV transmission. Much of the stigma literature in SSA suggests that women are widely regarded as vectors of HIV transmission (Dilger, 2003; Joffe & Bettega, 2003). Contrary to this, our informants overwhelmingly represented men as the vectors of HIV transmission, often through their involvement in what many children depicted as socially condemned behaviours (e.g. frequenting beer halls, sleeping with many women), and generally using their powerful position to exploit and abuse girls. South African children have also represented men as the vectors of AIDS in discussions of sexual violence (Bhana, 2009).

In this paper we have explored the representational field in which children’s responses to AIDS-affected children are negotiated. Some methodological factors deserve mentioning. Children were invited to draw a picture of a child whose family was affected by AIDS and subsequently asked to tell a story about this child’s life. In doing so, nearly half of the children drew a child caring for a bedridden parent – despite the fact that many PLWHAs (even in situations where access to ARVs is limited, as at the time of this study) are not permanently bedridden and are able to engage in domestic and income-generating activities between bouts of illness. Similar observations have been made by Gonzales-Rivera and Bauermeister (2007) in Puerto Rico.

Contrary to received wisdom about children’s drawings, our experience suggests that drawing, as a research methodology, may encourage stereotypical representations – rather than giving children opportunities to construct the more complex and nuanced types of accounts they gave in their written stories. Furthermore, in our informants’ drawings, many of the faces of AIDS-affected children had happy smiles despite accompanying stories representing the child’s lives as grim and exhausting. Our findings suggest that researchers should be cautious in using children’s drawings as a sole source of data, or in assuming that drawings are the best way to elicit frank accounts of emotional issues. Drawings should be complemented by an interview (draw-and-tell) or a written story (draw-and-write) to clarify the children’s own understandings of their drawings, and to open up space for children to communicate the nuances and contradictions that characterise their understandings of complex situations.

Furthermore, the content and tone of pictures and drawings tended to vary in our study. In general, drawings were more likely to focus on children as agents (caregivers, with positive attitudes and access to resources), whereas stories were more likely to focus on the emotional and negative circumstances of AIDS-affected children (upset, despair, bullying). Much remains to be learned about whether different media of communication (in our case drawings versus written essays and verbal stories) tend to generate more or less stereotypical representations, and positive or negative content.

There is a large developmental psychology literature that focuses on how children’s representations vary from one developmental stage to another (Piaget, 1929; Vygotsky & Cole, 1978). In our study we focused on children aged 10–12. As a follow-up to this study, we are keen to investigate how a wider range of age groups represent HIV/AIDS.

We have found that children’s combined drawings and essays add a useful and more complex dimension to understandings of AIDS stigma amongst children – in a field previously dominated by questionnaires, and by theoretical frameworks that focus on factors driving stigma, which in the process neglect factors that may mitigate AIDS stigma. There is a need for observational and long-term studies to unpick the complex relationship between individual and environmental factors that drive and mitigate children’s stigma of other children.

## Conclusion

We set out to explore children’s representations of AIDS and AIDS-affected children to further our understandings of whether and how children stigmatise AIDS-affected children, and to identify resources for stigma-reduction programmes. In line with our view that effective anti-stigma interventions are those that provide social spaces in which contrasting views and experiences can be debated, we have identified a diverse range of representations of AIDS-affected children. Whilst they were overwhelmingly portrayed as rejected, over-burdened and tragic figures, they were also presented as deserving of respect and compassion. Our informants also admiringly reflected how AIDS-affected children were able to negotiate support and help in dealing with their burdens, and actively participate in sustaining their households and providing care for their ailing parents.

We have no desire to downplay the degree of stigmatisation of AIDS-affected children, or to fail to acknowledge the immensity of their associated suffering. However we do believe that the co-existence of more positive representations of AIDS-affected children alongside stigmatising ones – even amongst a minority of children – serves as a promising potential resource for stigma-reduction programmes. Ideally such programmes would go beyond simply promoting HIV awareness through information provision, to facilitating social spaces in which groups of children could engage in critical thinking and dialogue (cf. Vaughan, 2010) regarding different ways of thinking about the stigmatised situation of their AIDS-affected peers. Such dialogue could serve as the first step for children’s co-construction of more positive and supportive representations, through the process of acknowledging and developing elements of positive thinking that exist alongside more negative ones. Social psychologists argue that programmes that enable people to identify and debate alternative ways of making sense of their social worlds open up promising arenas for the questioning and reframing of stigmatising social representations (Campbell, Nair, Maimane, & Nicholson, 2007). This is particularly the case when such debate is rooted in positive ways of thinking that already exist within the group (albeit often in an implicit and fragmentary way, or only amongst a minority of group members) that may need to be brought to people’s awareness before being used as a resource for challenging more negative views.

There are various compelling reasons for targeting stigma-reduction efforts at children, not only to reduce the suffering of AIDS-affected children, but also as an investment in the long-term challenge of reducing stigma amongst all age groups. Anxieties relating to sex and sexuality have been identified as key drivers of AIDS stigma (Campbell, Foulis, Maimane, & Sibiya, 2005). As argued above, pre-teenage children who are not yet sexually active may be less powerfully subject to the emotions and anxieties, or the symbolic taboos, frequently associated with mature sexual activity. As such, the stigmatising representations held by children may be more malleable and open to contestation and transformation than adult stigma. Furthermore, the admiration that *did* exist for AIDS-affected children in our study was related to the respect our informants held for other children’s caring roles. A study of AIDS care in a rural community in South Africa found that the activity of caring for people with AIDS was undervalued by adults, more particularly adult men (Campbell et al., 2009). If respect for caring is indeed a key resource for challenging stigma, it may have the most resonance with children who are more likely to understand the high value of caring than adults, who may have forgotten the feelings of dependency that characterise many aspects of childhood.

Schools have been identified as key arenas for stigma-reduction programmes (Brown, Macintyre, & Trujilo, 2003). Schools are uniquely placed to offer supportive social spaces that provide and mobilise support as well as allow teachers and children to promote anti-discriminatory behaviours (Kendall & O’Gara, 2007). Schools also have the potential to creatively involve pupils in sexual and health education (Hoadley, 2007). Such education could go ‘beyond awareness’ to tackle some of the negative and sometimes irrational or inaccurate understandings of AIDS and AIDS-affected children that mediated some of the negative representations held by children in our study. As the education system has the largest institutional network in many countries, school-based stigma-reduction programmes, using dialogical approaches to facilitate debate about alternative ways of making sense of the world, offer a unique opportunity for programmes seeking to reduce the bullying and stigmatisation of AIDS-affected children and PLWHA.

## Figures and Tables

**Table 1:** Coding framework: From codes to organising themes.

Global theme: Children’s representations of AIDS-affected children					
Codes	Frequency in stories	Frequency in drawings	Issues discussed	Basic themes	Organising themes
- AIDS Knowledge	6		• Biological impact	Knowledge of AIDS	Representations of AIDS
- ARVs help recovery	5	2	• Using gloves		
- AIDS contagious	4		• Fear of catching AIDS		
			• Nutritional needs		
			• ARV impact		

- AIDS through ‘bad’ behaviour	15	3	• Prostitutes, gifts for sex	Attitudes towards PWA	
			• Drinking, beer halls		
- Modes of transmission	9		• Promiscuity		
			• Men infecting women/girls		
- Representations of risk groups	6		• Mother to child transmission		
			• Unsafe sex		
- Death part of everyday life	14		• Sleeping all day	Outcomes of AIDS	
	3	23	• Weak, tired		
- PWA are bedridden and unproductive			• Messy compound		
	3	4	• Minimal body washing		
- PWA are messy			• Bereavement		

- Educational impact	15		• Begging, hunger	AIDS-affected children have no childhood, subject to abuse	Negative representations of AIDS-affected children
- Too busy doing care, household work	10	11	• Lack of concentration		
- Children affected by poverty	8	3	• School absence		
- Lost childhood	6		• No time to play		
- Vulnerable to abuse	6		• Too much inappropriate care		
			• Doing adult duties		
			• Rape and abuse		

- Children upset	15	4	• Often crying	AIDS-affected children experience psychosocial distress	
- Despair	5	2	• Unmanageable circumstances		
- Children lonely	4	1	• Worry about parents well-being		
- Children worry	3		• Too busy working to be with friends		
- Treated badly by peers	13	1	• Gossip	AIDS-affected children are ostracised and kept at distance	
- Negative reactions to parental HIV status	9		• Abandonment		
			• Lack of support		
			• Teasing of child		
			• Fear of touching the child		
			• Children won’t play with AIDS-affected child		

- Peer friendships	8	1	• NGOs, VCT centres	AIDS-affected children receive support from others	Positive Representations of AIDS-affected children
- Community support	7	1	• Supportive neighbours		
- Extended family support	5	1	• Supportive aunts and uncles		
- External support	2		• Friends support AIDS-affected children		
			• Play and fun, break from work		
- Children help caring for parents	12	6	• Feeding and washing	AIDS-affected children are caring and sustain their livelihoods	
- Children engage in IGA	7	6	• Administering drugs		
- Children help with domestic duties	4	4	• Cooking, cleaning, fetching water/firewood		
- Access to resources		14	• Head-of-house responsibilities		
			• Generating income		

- Children keep spirits high	5	7	• Children doing good work	AIDS-affected children are good children who deserve respect	
- Children with good qualities	5		• Learn new skills and knowledge		
- Working for social change	2		• Can educate others		
- Blessings from God	2		• Make world a better place		
			• Children kind and cheerful		
